# Robust light–dark patterns and reduced amyloid load in an Alzheimer’s disease transgenic mouse model

**DOI:** 10.1038/s41598-020-68199-5

**Published:** 2020-07-10

**Authors:** Rohan Nagare, Bernard Possidente, Sarita Lagalwar, Mariana G. Figueiro

**Affiliations:** 1 Lighting Research Center, Rensselaer Polytechnic Institute, Troy, NY USA; 2 Department of Biology, Skidmore College, Saratoga Springs, NY USA; 3 Neuroscience Program, Skidmore College, Saratoga Springs, NY USA

**Keywords:** Circadian rhythms and sleep, Alzheimer's disease

## Abstract

Circadian disruption resulting from exposure to irregular light–dark patterns and sleep deprivation has been associated with beta amyloid peptide (Aβ) aggregation, which is a major event in Alzheimer’s disease (AD) pathology. We exposed 5XFAD mice and littermate controls to dim-light vs. bright-light photophases to investigate the effects of altering photophase strength on AD-associated differences in cortical Aβ42 levels, wheel-running activity, and circadian free-running period (tauDD). We found that increasing light levels significantly reduced cortical Aβ42 accumulation and activity levels during the light phase of the light:dark cycle, the latter being consistent with decreased sleep fragmentation and increased sleep duration for mice exposed to the more robust light–dark pattern. No significant changes were observed for tauDD. Our results are consistent with circadian pacemaker period being relatively unaffected by Aβ pathology in AD, and with reductions in cortical Aβ loads in AD through tailored lighting interventions.

## Introduction

Recent evidence from mouse and human studies suggests that disruption of circadian rhythms and fragmentation of daily sleep–wake cycles are not only a consequence of Alzheimer’s disease (AD) progression but may also drive disease pathology and precede symptom onset^1–4^. Sleep disruption is also one of the principal reasons for the institutionalization of persons with AD^5^. The sleep–wake cycle is governed by 2 processes, the homeostatic system, which is associated with time awake, and the circadian system, which sets the timing of the sleep–wake cycle by sending an alerting signal, typically between 30 and 45 min post-awakening, and a sleeping signal with an increasing strength starting 2 h prior to the habitual sleep time^6^.

Light delivered by the local solar cycle is the dominant environmental factor that synchronizes the endogenous circadian clock in mammals, located in the suprachiasmatic nucleus (SCN) within the brain’s hypothalamus. Photic information, detected by the retinal photoreceptors (i.e., rods, cones, and intrinsically photosensitive retinal ganglion cells), is transmitted to the SCN via the retinohypothalamic tract for subsequent downstream behavioral, physiological, and metabolic processes. Disruption of the SCN resulting from exposure to irregular light–dark (LD) patterns or to insufficient light during the daytime (i.e., during the photophase of the daily LD cycle) leads to sleep disruption, which has been associated with beta amyloid peptide (Aβ) aggregation^7,8^. In fact, the alteration of sleep patterns may be an early event in AD that occurs years before clinical manifestations of the disease^3,9,10^.

In the present study, we worked with 5XFAD mice, a transgenic mouse model of AD that overexpresses mutant human amyloid beta precursor protein (APP695) with the Swedish (K670N, M671L), Florida (I716V), and London (V717I) familial Alzheimer's disease (FAD) mutations, along with human PS1 harboring 2 FAD mutations, M146L and L286V. As a result, the 5XFAD mouse exhibits early and robust AD pathology including production of intraneuronal Aβ at 1.5 months of age, a high Aβ42:Aβ40 ratio, plaque formation beginning at 2 months of age, and memory deficits^11^.

**Figure 1:**
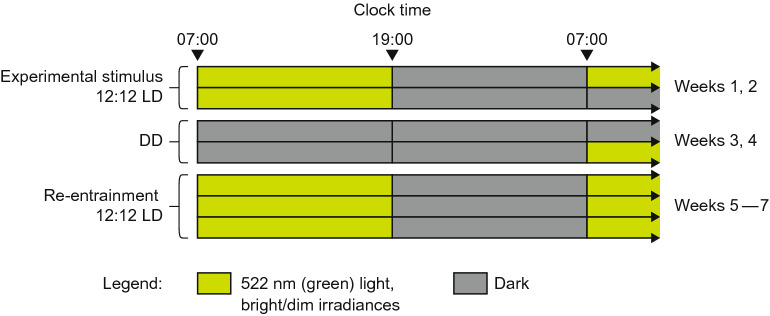
The experimental protocol for mouse Groups 1 and 2. The irradiance levels for the bright and dim experimental conditions are provided in Table 1. During re-entrainment, the mice were exposed to the same treatment photophase irradiances they experience during the initial 2 weeks of experimental 12:12 LD pattern; DD, 24 h of dark.

We performed a longitudinal assay of circadian locomotor activity, from approximately 20 weeks to 50 weeks of age, to characterize progressive changes in the endogenous circadian clock period and entrained (12:12 LD cycle) vs. free-running (constant darkness) circadian patterns of wheel-running activity. We exposed 5XFAD mice and littermate controls to dim-light vs. bright-light photophases (four 7-week cycles within each condition, consisting of 2 weeks of entrainment to a 12:12 LD cycle, followed by 2 weeks of constant dark, in turn followed by 3 weeks of re-entrainment to the 12:12 LD cycle) to investigate the effects of altering photophase light level (i.e., light level during the light portion of the daily LD cycle) on AD-associated differences in cortical Aβ42 levels, entrained and free-running patterns of wheel-running activity, and circadian free-running period (tauDD) (Fig. 1). It was hypothesized that 5XFAD mice subjected to the dim-light photophase would exhibit significantly higher cortical Aβ42 levels and increased daytime wheel-running activity compared to the 5XFAD mice experiencing the bright-light photophase. We expected the higher amplitude LD cycle to provide both a stronger entraining stimulus to the circadian clock and a more effective non-circadian suppression of activity during the photophase. We also expected these effects would reduce the disruption of sleep and, therefore, the clearance of beta-amyloid that is associated with AD. It was further hypothesized that tauDD would lengthen with age^12^, but that this lengthening would be less accentuated in the 5XFAD mice because it has been shown that the timing of the circadian clock is advanced in AD patients^13^.

## Results

### Amyloid accumulation (Aβ42)

**Figure 2:**
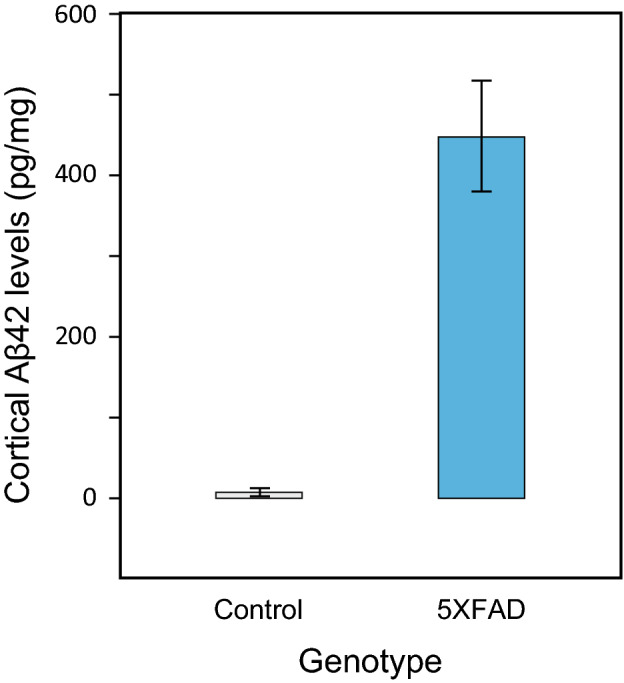
Cortical Aβ42 levels (pg/mg) in the 5XFAD mice and their littermate controls (assessment age = 343 days). There was a significant main effect of genotype on cortical Aβ42 levels. Reported mean values for each condition have been averaged across the rest of the independent variables. The error bars represent standard error of the mean.

All mice were euthanized for Aβ42 accumulation assessment at age 343 days. As expected, the univariate general linear model (GLM) employed in the analysis revealed a significant main effect of genotype on cortical Aβ42 levels (*F*_*1,34*_ = 58.8, *p* < 0.001, *η*_*p*_^*2*^ = 0.63), wherein cortical Aβ42 levels were significantly greater in the 5XFAD mice (mean = 447.7 pg/mg [SD = 299.1]) compared to the littermate controls (mean = 6.5 pg/mg [SD = 22.3]) (Fig. 2). Greater concentrations of Aβ42 were measured in females (mean = 228.3 pg/mg [SD = 360.0]) compared to males (mean = 183.9 pg/mg [SD = 227.4]), but the difference was not statistically significant (*F*_*1,34*_ = 1.4, *p* = 0.25, *η*_*p*_^*2*^ = 0.04).

**Figure 3:**
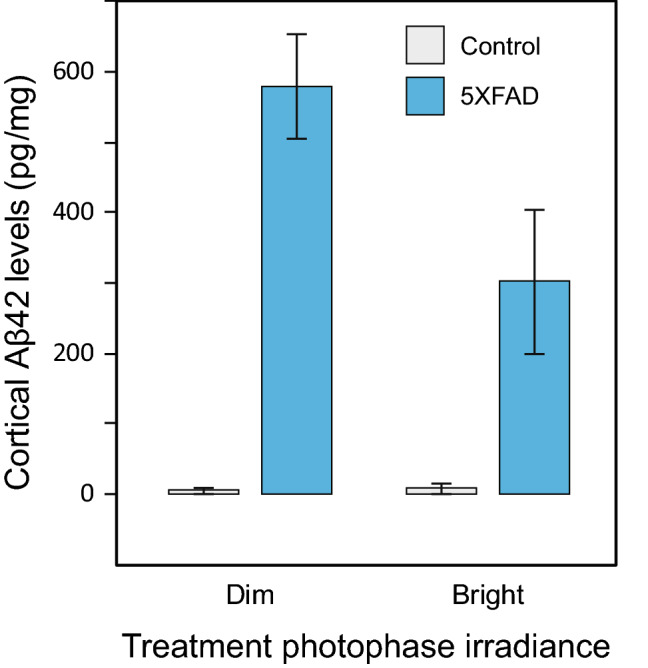
Significant interaction between the main effects of genotype (control vs. 5XFAD) and treatment photophase irradiance (dim vs bright) on cortical Aβ42 levels (pg/mg) (assessment age = 343 days). In the 5XFAD mice, levels of cortical Aβ42 were significantly higher in those exposed to dim light than in those exposed to the bright light, while there was no significant difference in cortical Aβ42 levels in the control group. Reported mean values for each condition have been averaged across the rest of the independent variables. The error bars represent standard error of the mean.

More interestingly, there was also a significant main effect of treatment photophase irradiance on amyloid load (*F*_*1,34*_ = 5.3, *p* = 0.03, *η*_*p*_^*2*^ = 0.14), wherein significantly lower cortical Aβ42 levels were found in mice treated with the bright-light condition (mean = 133.9 pg/mg [SD = 245.1]) compared to those treated with the dim-light condition (mean = 278.2 pg/mg [SD = 333.6]) during the 2-week experimental exposures. A significant interaction between the main effects of treatment photophase irradiance and genotype (*F*_*1,34*_ = 5.5, *p* = 0.03, *η*_*p*_^*2*^ = 0.14) was also observed (Fig. 3), wherein the Aβ42 levels for the control mice were quite close to zero, irrespective of the light’s strength (dim vs. bright). No other interactions were statistically significant (*p* > 0.05).

### Photophase activity

**Figure 4:**
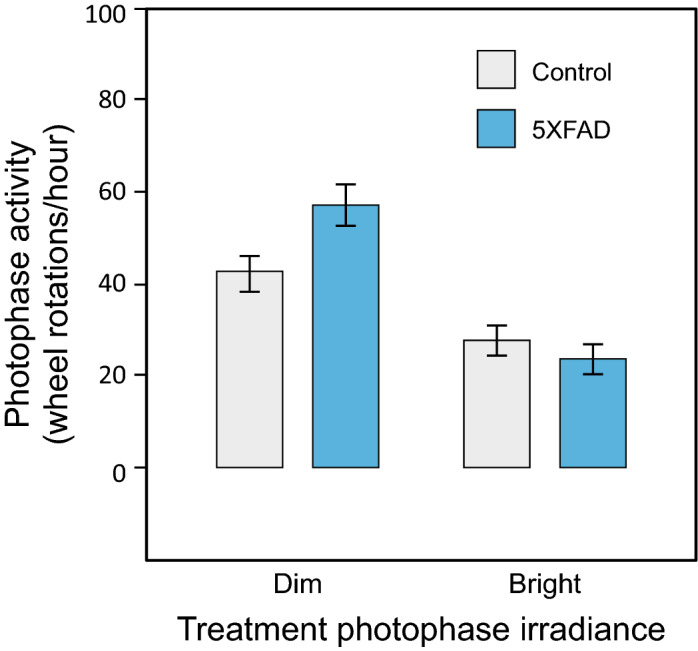
Mean photophase activity with respect to treatment photophase irradiance and genotype (pooled for sex). A significant interaction between the main effects of treatment photophase irradiance and genotype on photophase activity was observed. The error bars represent standard error of the mean.

The univariate GLM did not reveal statistically significant effects of genotype and sex for activity (i.e., wheel running) during the photophase. During the photophase, the mean number of wheel rotations per hour was 39.8 (SD = 31.2) and 35.1 (SD = 26.0) for the 5XFAD and control mice, respectively, and 35.0 (SD = 23.6) and 39.7 (SD = 32.9) for males and females, respectively. The GLM revealed a significant main effect of treatment photophase irradiance on photophase activity (*F*_*1,38*_ = 25.5, *p* < 0.001, *η*_*p*_^*2*^ = 0.40), wherein significantly lower activity levels during the light phase were recorded in mice treated with the bright light condition (mean = 25.6 [SD 22.3]) compared to those treated with the dim light condition (mean = 49.5 [SD 29.6]) during the 2-week experimental exposures. A significant interaction between the main effects of treatment photophase irradiance and genotype was also observed (*F*_*1,38*_ = 4.8, *p* = 0.04, *η*_*p*_^*2*^ = 0.11), wherein the suppression of photophase activity was most pronounced in 5XFAD mice under the bright light condition (Fig. 4). No other interactions were statistically significant (*p* > 0.05).

### Free-running period (tauDD)

The univariate GLM did not reveal statistically significant effects of genotype, sex, or treatment photophase irradiance on tauDD. Mean tauDD was 23.59 h (SD = 0.34) and 23.76 h (SD = 0.28) for the 5XFAD and control mice, respectively; 23.67 h (SD = 0.38) and 23.68 h (SD = 0.25) for males and females, respectively; and 23.68 h (SD = 0.36) and 23.67 h (SD = 0.26) for mice pre-treated with the dim and bright conditions, respectively. There was no significant effect of tauDD on age.

## Discussion

The present study investigated the effect of longitudinal alteration of the photophase light level (i.e., dim vs. bright light level during the light portion of the daily LD cycle) on cortical Aβ42 accumulation, photophase activity, and circadian free-running period (tauDD) in a 5XFAD transgenic mouse model. One of the principal and novel findings was that increasing the narrow-band (peak wavelength = 522 nm) light stimulus’s irradiance in the light portion of the daily 12:12 LD pattern from 0.14 μW cm^−2^ to 3.41 μW cm^−2^ significantly reduced cortical Aβ42 accumulation, a hallmark of AD. Moreover, the results showed that exposure to a strong 12:12 LD pattern using the higher irradiance (bright) light, irrespective of genotype, significantly reduced activity levels during the photophase. These reduced activity levels are consistent with decreased sleep fragmentation and increased sleep duration for mice that were exposed to the more robust 12:12 LD pattern. More importantly, and as expected, the effect of receiving a strong 12:12 LD pattern was greater in the 5XFAD mice than in the controls. These results may lend significant support to a recent human study^14^ showing that exposing AD patients to much higher light levels than those typically found in the built environment improved measures of sleep, depression, and agitation. It may seem paradoxical that brighter light during the photophase of the LD cycle should have beneficial effects on both nocturnal and diurnal species. We propose that a common mechanism may be stronger entrainment of the light-entrainable pacemaker in the SCN, leading to a more effective non-circadian suppression of activity in the photophase in mice, and an enhanced daytime wakefulness in humans. Both diurnal and nocturnal circadian systems are programmed to entrain to the same natural LD cycles, so it is not unreasonable to assume that both species will benefit from exposures to robust LD cycles. In fact, studies with healthy office workers showed that increasing daytime light led to better nighttime sleep^15,16^.

To our knowledge, this is the first study to show a direct impact of photophase strength on cortical Aβ42 accumulation in an AD transgenic mouse model. A recent study by Iaccarino, et al.^17^ demonstrated that 40 Hz flickering light effectively decreased amyloid levels and plaque pathology in the visual cortex of a 6-month-old AD transgenic mouse model. Importantly, that study also showed that increasing gamma oscillations using 40 Hz optogenetic or visual stimulation induced gene expression changes and morphological transformation of microglia, with a significant increase in microglia cell body diameter. Finally, Iaccarino et al. showed that 40 Hz flickering light promoted microglia–Aβ interactions, resulting in less Aβ plaque accumulation.

Although objective sleep measures were not obtained in this study, the increase in wheel-running activity during the photophase among mice exposed to lower light levels suggests increased sleep fragmentation, which may help to explain the increased amyloid load. A diurnal Aβ pattern has been demonstrated and is believed to be related to higher neuronal activity during wakefulness and lower neuronal activity during sleep, especially slow-wave sleep^8,18^. Studies using animal models^19–21^ have shown that amyloid plaque aggregation can be modulated up or down by driving sleep deprivation or sleep induction, respectively. In humans, non-rapid eye movement, slow-wave activity sleep was found to be inversely associated with AD pathology^22^. Future animal studies should collect objective sleep data to determine whether sleep was indeed negatively affected by the weaker (i.e., dim light) 12:12 LD pattern.

Interestingly, the endogenous clock period was not affected by differences in photophase strength or genotype. It is likely that intact circadian clock function perhaps mediated the positive effect of the stronger (i.e., bright light) 12:12 LD pattern, indirectly reducing the cortical Aβ42 load in the 5XFAD mice.

The lack of sex differences with regard to Aβ42 load in the present study is inconsistent with previous studies involving 5XFAD mice and other strains^23–26^, although the results can be attributed to the older Aβ assessment age of the mice herein. For example, Oakley et al.^11^ reported higher Aβ42 levels in young females, up to 9 months of age, compared to that study’s age-matched males.

Age also had no significant effect on circadian free-running period in both the control and 5XFAD mice, which perhaps supports evidence that age effects on tauDD in mice can be strain-dependent^12^, and is consistent with an absence of an effect of AD pathology on circadian clock period in the 5XFAD mouse model.

It is also worthwhile to consider that during the 4 cycles of the study’s 7-week protocol, all mice experienced the same stimulus only for the 2 weeks of DD, since the following 3 weeks of 12:12 LD re-entrainment was accomplished using the same light–dark patterns that the mice received during the initial 2 weeks of the treatment 12:12 LD cycle. The group exposed to the bright light condition may have re-entrained more quickly and suffered less circadian disruption as a result. We also recognize that all the mice used in this study had access to running wheels during the 28 weeks of treatment and “wheel running” by itself was not employed as an independent experimental variable. Hence, we cannot separate any potential effect of voluntary wheel running from the photophase light level effects on the measured outcomes. Interestingly, a recent study by Robison, et al.^27^ involving a Tg-SwDI mouse model has shown that vascular amyloid load was not affected by long-term (4 to 12 months) voluntary wheel running. Further studies are needed to assess the effects of altering photophase strength alone on Aβ42 levels. It is likely that the observed reduction in Aβ42 loads between mice treated to the bright-light and dim-light conditions occurred cumulatively from the start of the treatment. However, it is not possible to infer whether the observed reduction in the Aβ42 load in mice treated to bright light level photophases was due to prevention of amyloid accumulation or reversing deposition of accumulated Aβ in proportion to the treatment^28^.

It would be useful for further studies to assay Aβ levels and plaque morphology in additional areas of the brain, determine whether Aβ plaque pathology was affected, explore additional light treatment protocols, and to include other Aβ and tauopathy mouse AD models with direct assays of sleep quality.

## Conclusions

The present results are consistent with circadian pacemaker period being relatively unaffected by Aβ pathology in AD, and with reductions in cortical Aβ loads in AD through bright light treatment. Further research on bright light treatment is essential for exploring possible applications for AD and other forms of dementia in humans.

## Methods

### Animal model

This study utilized 23 5XFAD mice (34840-JAX, The Jackson Laboratory, Bar Harbor, ME; https://www.jax.org/strain/006554) and 24 littermate controls, optimally balanced for sex ratio within genotypes (12 females per genotype), that were purchased at 35 days of age. All experimental procedures were approved by the Skidmore College Animal Care and Use Committee and conformed to international ethical standards^29^.

### Housing and lighting apparatus

All mice were individually housed during the experiment in *STARR Life Science Incage* running wheel cages (11 in. × 7 in. × 5 in.[L × W × H]), each furnished with a 4.5-in.-diameter wheel, 0.125-in. crushed corncob bedding, and a 2 g Nestlet cotton square. Food (Prolab RMH 3,000 Lab Diet pellets) and tap water were provided ad libitum. Throughout the experiment, the mice were maintained under a controlled ambient temperature of 71 ± 2 °C.

**Figure 5:**
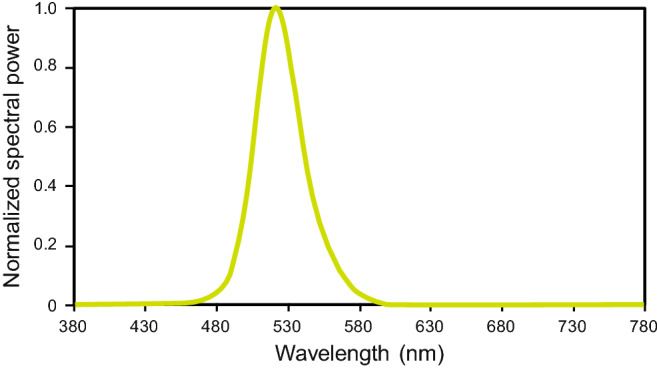
Normalized spectral power distribution for the experimental light stimulus. The spectral power of the narrow-band (peak wavelength = 522 nm) green LED. Source was measured via spectrometer (Model USB650 Red Tide Spectrometer, Ocean Optics, Winter Park, FL).

The light stimulus for the mice was provided and controlled using a custom-built light-emitting diode (LED) system that was installed in each cage. Linear strip lights of narrow-band (peak wavelength = 522 nm) green LEDs (iCove, ColorKinetics, Detroit, MI) were used to generate 2 experimental treatment photophase irradiances, bright and dim, each with its own discrete irradiance level as measured horizontally on the cage floors (Table 1). The light source’s normalized spectral power distribution is shown in Fig. 5. The irradiance level for the bright condition was greater than the absolute threshold (~ 1 μW cm^−2^) required to induce a circadian phase shifting response but lower than that for response saturation (~ 10 μW cm^−2^), following a dose–response function for green light stimulus developed by Bullough and colleagues for *Mus musculus*^30,31^.

**Table 1:** Photometric characteristics of the green light stimulus.

Experimental condition	Mean ± SEM irradiance (μW cm^−2^)	Mean illuminance (lux)	Mean photon flux (photons m^−2^ s^−1^)
Dim	0.14 ± 0.01	0.70	3.7e + 16
Bright	3.41 ± 0.47	16.80	90.3e + 16

### Protocol

All mice were acclimated and aged for 104 days in individual cages housed within the laboratory facility in Skidmore college, Saratoga Springs, NY, under a constant 12:12 LD pattern (3500 K fluorescent lamps, irradiance of approximately 70 μW cm^−2^) prior to commencing the experimental protocol. Following acclimatization, the mice were divided into 2 groups optimally balanced across genotype and sex: Group 1 (23 individuals, 11 5XFAD, 12 females) and Group 2 (24 individuals, 12 5XFAD, 12 females). Group 1 was assigned to the dim condition and Group 2 was assigned to the bright condition.

Over the course of the experiment, all mice underwent a looped 7-week experimental protocol (see Fig. 1) starting at age 139 days and ending at age 313 days. (Four cycles of the looped protocol were run throughout the course of the study). The first 2 weeks of each cycle presented the experimental stimulus, wherein the irradiance for the light portion of the 12:12 LD pattern was pre-set to either the dim or bright settings shown in Table 1. Irradiance levels were verified periodically via spectrometer (Model USB650 Red Tide Spectrometer, Ocean Optics, Winter Park, FL).

The 12:12 LD pattern was followed, without interruption, by a 2-week continuous dark (DD) pattern to permit estimates of free-running period (tauDD). Wheel-running data were downloaded using Vitalview Data Acquisition Software (Starr Life Sciences, Oakmont, PA) and tauDD was estimated using chi-square periodogram (Rhythmwatch software, Minimitter Corporation, Bend, OR). The final 3 weeks of each 7-week cycle served the purpose of re-entraining the mice, following a 12L:12D pattern employing the same light source used during the 2 weeks of 12:12 LD treatment to establish a common phase prior to application of the next 12:12 LD experimental stimulus. Following the DD period (weeks 3 and 4) of the fourth and final experimental cycle, the mice remained in the same lighting used for re-entrainment until they were euthanized for Aβ42 accumulation assessment at age 343 days.

### Cortical Aβ42 ELISA

Cortical tissue was harvested at 49 weeks of age, and prepared according to the protocol established for 5XFAD mice by Oakley, et al.^10^. Specifically, following extraction, the tissue was flash frozen and re-suspended in 3 to 4 volumes of PBS-0.5% Triton supplemented with protease inhibitors (A32965 ThermoFisher, Waltham, MA). Tissue was homogenized with a Corning Dounce homogenizer (1234F35 Thomas Scientific, Swedesboro, NJ), freeze–thawed 3 times, and cleared at 15,000 rpm for 15 min at 4 °C. Cleared homogenates were supplemented with guanidine hydrochloride to a final concentration of 5 M to solubilize plaques. To detect human Aβ42 levels, cleared homogenates were diluted in Standard Diluent Buffer and measured by the Invitrogen Human Aβ42 ELISA kit (KHB3441, ThermoFisher, Waltham, MA) following manufacturer’s instructions. Final guanidine hydrochloride concentrations were under 0.1 M. All samples were run in duplicate; their readings fell within the range of the standard dose responses curve. Total protein was measured by a protein assay kit (5000002, Bio-Rad, Hercules, CA) and used for normalization.

### Statistical analysis

Statistical analysis was performed with SPSS statistical software (SPSS version 25, IBM, Armonk, NY, USA). When reporting a significant main effect of an independent variable (e.g., genotype), the responses for the dependent variable were averaged across all other independent variables. For all statistical analyses, the between-subjects fixed factor genotype contained 2 levels (control and 5XFAD). The between-subjects fixed factor sex contained 2 levels (male and female). The between-subjects fixed factor photophase strength contained 2 levels (dim and bright). Results were considered significant if the resulting *p* values were < 0.05.

#### Amyloid (Aβ42) accumulation

The univariate GLM for Aβ42 accumulation employed 3 between fixed factors (genotype, sex, treatment photophase irradiance) and amyloid load (Aβ42 levels) as the dependent variable. Aβ42 data could not be estimated for 4 mice that died during the experiment. Preliminary statistical analysis detected the Aβ42 value for one control mouse as an extreme outlier that was excluded from the analysis.

#### Photophase activity levels

The univariate GLM for photophase activity levels employed 3 between-subjects fixed factors (genotype, sex, treatment photophase irradiance), with photophase wheel-running activity levels (wheel rotations/hour) as the dependent variable, to provide estimates of sleep fragmentation. The analysis of photophase activity levels was limited to the data obtained during exposure to the experimental stimulus (weeks 1 and 2), and the data were averaged across all four 7-week experimental cycles.

#### Free-running period (tauDD)

Calculations of tauDD were based on wheel-running activity data. The univariate GLM for tauDD employed one within-subjects factor (age) and 3 between-subjects fixed factors (genotype, sex, treatment photophase irradiance) with tauDD values as the dependent variable. The within-subjects fixed factor age contained 4 levels corresponding to the four 2-week DD cycles (Assessment age: 167, 216, 265, 313 days). Preliminary statistical analysis did not reveal any outliers.

## Data Availability

The data that support the findings of this study are available from the corresponding author upon reasonable request.
